# Longer hospital stay is associated with higher rates of tuberculosis-related morbidity and mortality within 12 months after discharge in a referral hospital in Sub-Saharan Africa

**DOI:** 10.1186/1471-2334-14-409

**Published:** 2014-07-22

**Authors:** Nicola M Zetola, Nenad Macesic, Chawangwa Modongo, Sanghuk Shin, Ronald Ncube, Ronald G Collman

**Affiliations:** 1 Division of Infectious Disease, University of Pennsylvania, Philadelphia, Pennsylvania, USA; 2 University of Botswana Medical School, University of Botswana, Gaborone, Botswana; 3 Botswana-UPenn Partnership, Gaborone, Botswana; 4 Division of Infectious Diseases, University of Melbourne, Melbourne, Australia; 5 School of Medicine, University of California, Los Angeles, USA; 6 Botswana National TB Program, Gaborone, Botswana; 7 Division of Pulmonary and Critical Care Medicine, University of Pennsylvania, Philadelphia, Pennsylvania, USA

**Keywords:** Tuberculosis, Transmission, Nosocomial, Infection control, HIV, Morbidity, Mortality

## Abstract

**Background:**

Nosocomial transmission of pulmonary tuberculosis (PTB) is a problem in resource-limited settings. However, the degree of TB exposure and the intermediate- and long-term morbidity and mortality of hospital-associated TB is unclear. In this study we determined: 1) the nature, patterns and intensity of TB exposure occurring in the context of current TB cohorting practices in medical centre with a high prevalence of TB and HIV; 2) the one-year TB incidence after discharge; and 3) one-year TB-related mortality after hospital discharge.

**Methods:**

Factors leading to nosocomial TB exposure were collected daily over a 3-month period. Patients were followed for 1-year after discharge. TB incidence and mortality were calculated and logistic regression was used to determine the factors associated with TB incidence and mortality during follow up.

**Results:**

1,094 patients were admitted to the medical wards between May 01 and July 31, 2010. HIV was confirmed in 690/1,094 (63.1%) of them. A total of 215/1,094 (19.7%) patients were diagnosed with PTB and 178/1,094 (16.3%) patients died during the course of their hospitalization; 12/178 (6.7%) patients died from TB-related complications. Eventually, 916 (83.7%) patients were discharged and followed for one year after it. Of these, 51 (5.6%) were diagnosed with PTB during the year of follow up (annual TB rate of 3,712 cases per 100,000 person per year). Overall, 57/916 (6.2%) patients died during the follow up period, of whom 26/57 (45.6%) died from confirmed TB. One-year TB incidence rate and TB-associated mortality were associated with the number of days that the patient remained hospitalized, the number of days spent in the cohorting bay (regardless of whether the patient was eventually diagnosed with TB or not), and the number and proximity to TB index cases. There was no difference in the performance of each of these 3 measurements of nosocomial TB exposure for the prediction of one-year TB incidence.

**Conclusion:**

Substantial TB exposure, particularly among HIV-infected patients, occurs in nosocomial settings despite implementation of cohorting measures. Nosocomial TB exposure is strongly associated with one-year TB incidence and TB-related mortality. Further studies are needed to identify strategies to reduce such exposure among susceptible patients.

## Background

Tuberculosis remains one of the major public health problems worldwide [1,2]. This high burden of disease together with the increasing prevalence of resistant strains highlights the need to maximize infection control measures in settings in which TB transmission is likely occurring [3,4]. Nosocomial outbreaks of TB have been well documented [5-9]. In areas were HIV and TB are highly prevalent, health care settings select for HIV patients with advanced disease, usually admitted for the treatment of infectious complications, who may be highly susceptible to TB infection and disease, as well as TB patients with complicated disease, often highly infectious, that have failed outpatient treatment (e.g. severe cavitary disease, extensive, multi-lobar disease and drug-resistant TB). Therefore, nosocomial acquisition of TB among HIV-infected and other susceptible patients is believed to be a major source of morbidity and mortality and may significantly contribute to the spread of disease in the community [5].

One widely used strategy, advocated as an alternative to individual isolation, is the cohorting of patients admitted with confirmed or suspected TB (“TB bays”) [10]. However, despite widespread use worldwide, the impact of this approach on TB exposure, the intensity of the exposure or, most importantly, transmission in settings with high prevalence of HIV and TB remains unclear [3,4,11]. A major determinant of the effectiveness of cohorting is the performance of the triage approach on admission and the recognition of suspect cases during the hospitalization period. In endemic settings several factors make the situation particularly complicated [5]. First, clinical triage, the most widely used approach for the triage of patients with suspicion for TB worldwide, has poor sensitivity and specificity in identifying patients with TB [12]. Recently, new diagnostic tools with rapid turn-around and greater sensitivity and specificity that may allow appropriate triage of such patients to cohorting wards have become available (e.g. GeneXpert MTB/RIF, Cepheid Inc.) [13-16]. However, these diagnostic tools are not yet widely available in resource-limited settings. Further, even if these new diagnostic tools allow a perfect sensitivity and specificity for the triage of patients with active TB to the appropriate “TB bay”, cohorting these patients (who have already demonstrated themselves as predisposed to develop active TB) together with multiple highly infectious patients may even lead them to acquire infections with multiple TB strains.

Determining TB-related outcomes and the factors associated with them among patients following discharge from settings of potential nosocomial transmission is crucial to better understand the potential impact of nosocomial TB in epidemics, and to inform patients, healthcare workers and policy makers [17-23]. In addition, little is known regarding outcomes of hospitalized TB patients after discharge [10]. TB-infected patients admitted to the hospital tend to have more serious forms of the disease. Thus, determination of the rates and outcomes of TB recurrence or relapse in the community (after patient’s discharge) among those who required hospitalization for TB also carries great clinical and public health importance.

Short-term outcomes, such as inpatient mortality and acute functional decline following hospitalisation, have been extensively studied and are well established for a number of medical illnesses (including TB) in both developed and developing country settings. However, few data are available on the intermediate- to long-term TB-related outcomes of patients admitted to the medical wards, especially in developing country settings where documentation and follow-up are problematic [10,24-31]. In this study we determined: 1) the nature, patterns and intensity of TB exposure occurring in the context of current TB cohorting practices in a major urban medical centre with a high prevalence of both TB and HIV; 2) the incidence of TB within the first year after discharge; and 3) the mortality associated with those one-year incident TB cases. Additionally, we determined comprehensive all-cause mortality for all patients admitted to the medical ward, including mortality during the patient’s inpatient stay and in the first year after discharge.

## Methods

### Settings

**Figure 1:**
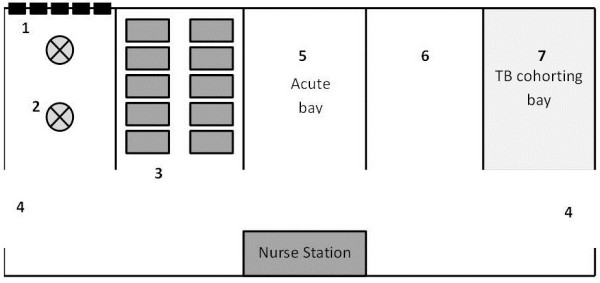
**Shows the schematic distribution of the bays and the tuberculosis cohorting bay within the medical ward.** Position and number of windows, fans and doors are represented as well. Each ward (male and female) is divided in 5, 10-bed bays (6). (1) Each bay has 5 windows and (2) 2 ceiling fans. (3) Beds are organized in two lines of 5 beds each, located on in front of the other. (4) Double doors are located at each extreme of the ward. (5) Traditionally, sickest patients are located in the bay in front of the nurse station. (7) The most distant bay, located next to the main back door is used as the TB cohorting bay.

All patients admitted to the medical wards between May 01 and July 31, 2010 at a large tertiary referral hospital in Botswana, were enrolled in this observational cohort. With a base population of approximately 350,000 persons, this hospital serves as the main referral centre for the Southern region of the country [32]. Wards are “open” (Figure 1) and divided according to the sex of the patients.

#### The medical wards

This study was conducted at the male and female medical wards which are, by far, the busiest wards in the hospital. At these wards, an average of 400 patients are cared for on a monthly bases (approximately 200 patients in each ward). Each ward is divided into 5 bays with a maximum capacity of 10 patients each (Figure 1). Four bays are used for general medical non-TB admissions, while the fifth one is used for the cohorting of patients with suspected or confirmed active pulmonary TB (Figure 1).

#### Infection control measures in place

Our hospital follows the guidelines set out in the national TB exposure control guidelines [33]. The TB infection control guidelines are based on the World Health Organization (WHO) recommendations and are based on a three-level hierarchy of control measures (refer to Additional file 1) [10].

#### Administrative measures

Administrative measures include triaging and cohorting of patients suspected to have TB, intensified TB case finding among patients with symptoms and risk factors for TB, early initiation of antituberculous treatment (ATT), and early discharge of patients infected with TB among others.

#### Use of respiratory protective equipment

N95 masks required and are used virtually by all health practitioners during direct contact with patients in the TB cohorting ward. Surgical masks are provided to the all patients with diagnosis or suspicion for TB and their use at all times is encouraged.

### Data collection

Data were collected on standardized forms on a daily basis. Every day, nurses reassess the patients’ diagnosis and clinical status as well of cubicle occupancy and staffing and patients relocated accordingly. As most of such patient relocation occurs during the morning nursing shift, all data were consistently collected at the same time of the morning and evening, after the patient re-location was finalized. The number of patients per cubicle, their position, environmental factors was collected at this time. More specifically, the following variables were collected from the medical records:

#### Demographics and infectiousness of the source/index case

Basic demographics, reason from admission, working diagnosis, admission date, history of TB prior to the current episode (yes/no), history of prior ATT (yes/no and dates). For patients under evaluation for TB, information on the direct observed therapy site and sputum collection was recorded. Whether the patient was suspected to have TB was recorded as not suspected or suspected. Patients with known TB on admission who were on ATT were also considered susceptible for reinfection, relapse or recurrence. All their information was recorded as well. The diagnosis of TB at discharge was assessed and categorized as no TB, suspected TB or confirmed TB. Patients were considered to have confirmed TB at discharge if they had at least one sputum smear positive for acid-fast bacilli (AFB) obtained during their hospitalization. AFB status and ATT status of patient was carefully recorded. In addition, we followed the location of each patient in the ward (twice a day) and his/her proximity to patients with known PTB.

#### ATT initiation during hospitalization was noted

Patients with suspected pulmonary TB (PTB) at the time of discharge were followed at their referral clinics to verify whether the diagnosis of TB was confirmed. Patients with positive AFB smears or cultures were considered as confirmed PTB. Patients that failed to improve with standard pneumonia treatment but that improved on ATT were considered to have clinically confirmed PTB.

#### Identification of missed diagnosis

In addition, our study database was matched with the one from the National TB Reference Laboratory to identify missed diagnosis of PTB during the hospitalization period. These missed diagnosis were defined as cases in which PTB was not suspected on admission or discharge or cases in which PTB was suspected on admission but not on discharge but it was confirmed within 3 months after discharge (by self-referral of the patient to a TB clinic).

#### Severity of pulmonary disease

All patients with suspicion or confirmed PTB had a chest X ray. All X rays were read by a radiologist and an pulmonologist and an infectious diseases specialist; all with extensive clinical experience in the management of TB patients. Each X ray was divided horizontally by eye into 3 equal zones per lung field. The extent of disease was be determined by the number of zones containing lesions in the lung parenchyma (mild for one zone, moderate for two zones and severe for 3 zones).

#### Quantification of TB exposure

To determine how best to assess exposure to infectious PTB patients that may predict the development of active TB during the follow-up period after discharge; we developed a semi-quantitative index of overall TB exposure, and compared the performance of three measurements.

The total number of days admitted to the medical ward.

The total number of days admitted to the hospital divided in: a) days spent at the cohorting bay; versus, b) days spent at the general medical bay.

The third measurement attempted to quantify intensity of exposure by considering the number of adjacent TB patients and the number of non-adjacent TB cases in each bay, every day. To calculate the daily exposure index per participant per day, adjacent cases were given twice the weight of non-adjacent ones. For example, if a participant had 2 adjacent TB cases and one non-adjacent TB patient in his/her bay, the “exposure index” for that day would be calculated as: 2 × 2 + 1 = 5.

#### The immune status of the patients at risk

HIV status and CD4 cell counts were recorded on all patients with new and old HIV diagnosis. History of anti-retroviral therapy (ART) was collected on patients known to be HIV infected at the time of admission. As per routine practice, patients with history of negative or unknown HIV status were re-tested on admission.

### Follow up

Every 3 months for a total of 12 months, data from each enrolled patient were extracted from paper medical records and electronic databases at the hospital, TB clinics, Botswana National Tuberculosis Program, the Botswana National Tuberculosis Reference Laboratory, HIV clinics, and the Botswana National HIV Program. Additionally, the National Death Registry was searched for details about patient mortality, date of death, and hospital readmissions. Data abstraction was focused on the identification of new episodes of TB, episodes of TB recurrence or relapse, overall mortality and TB-associated mortality. Patients unable to be contacted by any of the above methods were considered ‘lost-to-follow-up’. For this analysis, ‘untraceable’ patients were considered to be alive since their names could not be found in the National Death Registry. Due to the observational nature of the design, patients were never contacted during the entire duration of the study.

### Definitions and outcomes

Given the different rates of progression to active PTB after the exposure of HIV-infected and HIV-uninfected patients, all analyses were performed separately by HIV infection status. Tuberculin conversion was not used as an outcome given the extremely high rates of positivity in our population and the almost universal exposure to TB among adults in our setting. Thus, our main outcomes were TB incidence, all-cause mortality and TB-associated mortality during the follow-up period. We used the World Health Organization (WHO) recommendation to define our TB cases [33,34]: *a) Case of TB:* A patient in whom TB was confirmed by bacteriology or diagnosed by a clinician. *b) Definite case:* A patient with positive culture for Mtb or a patient with two sputum smears positive for acid-fast bacilli (AFB+). *c) Pulmonary case:* A patient with tuberculosis disease involving the lung parenchyma. *d) Smear-positive pulmonary case:* A patient with one or more initial sputum smear examinations (direct smear microscopy) AFB+; or one sputum examination AFB + and radiographic abnormalities consistent with active pulmonary tuberculosis as determined by a clinician. *e) Smear-negative pulmonary case:* A patient with pulmonary tuberculosis not meeting the above criteria for smear-positive disease. Diagnostic criteria included: at least two sputum smear examinations negative for AFB; and radiographic abnormalities consistent with active pulmonary tuberculosis; and a decision by a clinician to treat with a full course of ATT; or positive culture but negative AFB sputum examinations. *f) Extrapulmonary case:* A patient with tuberculosis of organs other than the lungs (e.g. pleura, lymph nodes, abdomen, genitourinary tract, skin, joints and bones, meninges). Diagnosis was based on one culture-positive specimen, or histological or strong clinical evidence consistent with active extrapulmonary disease, followed by a decision by a clinician to treat with a full course of ATT. A patient in whom both pulmonary and extrapulmonary TB was classified as a pulmonary case. *g) New case:* A patient who never had treatment for tuberculosis. *h) Re-treatment case:* A patient previously treated for TB, who started on a re-treatment regimen after previous treatment had failed, who returned to treatment having previously defaulted, or who was previously declared cured or treatment completed and was diagnosed with bacteriologically positive TB (relapse).

### Statistical analyses

Descriptive statistics were determined by standard methods. Groups were compared using Fisher’s exact or Wilcoxon-rank-sum tests as appropriate. Odds ratios were calculated for all factors potentially associated with a final diagnosis of PTB. We controlled for possible confounding or effect-modifying factors by using logistic regression. We determined potential confounders a priori based on estimation of their significance as epidemiologic factors during the preliminary crude analysis (significant at *p* ≤ 0.05) and biological plausibility. Potential confounders included age, sex and prior TB history. For analyses restricted to those with HIV, we also evaluated the CD4 count and use of ART at baseline as potential confounders. Factors indicating severity of disease included semi-quantitative bacillary load (by AFB microscopy: scanty, 1+, 2+ and 3+) and chest X ray results. Potential confounders were considered actual confounders if their inclusion in the multivariable model changed the unadjusted odds ratio (OR) by 10% or more. In addition, given that our main measures of TB exposure were highly collinear, different models were built for each of those variables. Finally, in an attempt to distinguish patients with the highest probability of having a relapse of their prior TB disease from those who develop active disease due to a nosocomial exposure, we performed separate analyses for the total population and the population of patients who were not diagnosed with TB during their hospital admission. Two-sided *p*-value <0.05 was considered statistically significant. We used STATA/IC version 13.0 (StataCorp Inc, College Station, Texas) for the analyses.

### Ethics and human subject protection

The Human Research Committee of the Government of Botswana and the hospital’s Ethics Committee approved this study. The need for informed consent from the participant was waived by both Research Ethic Review Committees.

## Results

### Before admission to the medical ward

1,283 patients were scheduled for admission to the medical ward. However, 189 (6.8%) of them died at the Emergency Department before they could be transferred to the wards. 34 (18%) of these 189 patients had the working diagnosis of pneumonia. TB was retrospectively confirmed through positive AFBs or culture in 4 (12%) and remained as the most probable ethologic agent on 16 (47%) of those 34 cases of pneumonia (Additional file 2).

### During admission to the medical ward

1094 patients were admitted to the medical wards during the study period. Demographic, clinical and radiographic characteristics of these patients are shown in Tables 1 and 2. Additional file 2 shows the distribution of patients according to the place of admission (cohorted vs. not cohorted) and diagnosis of PTB. Of the admitted patients, 131 (12%) spent their entire hospital stay at the TB cohorting bay and an additional 80 (7.3%) spent part of their stay in the TB cohorting bay (Additional file 2). The mean number of days of hospitalization was 7.8 (standard deviation [SD] 6.6) for the entire population, 7.3 (5.4) for HIV-uninfected and 8.2 (7.2) for HIV-infected (Table 3). During the hospitalization period, 198 (18%) died. PTB was diagnosed in 164 of the 896 patients (18%) who were eventually discharged.

**Table 1:** Overview of study features and admission characteristics of 1,094 participants

		**Total**	**Cohorted**	**General bay**
		**n = 1,094**	**n = 211**	**n = 883**
Age	18 – 29 years	298 (27.2%)	53 (25.1%)	245 (27.7%)
	30 – 39 years	282 (25.8%)	56 (26.5%)	226 (25.6%)
	40 – 49 years	193 (17.6%)	40 (19.0%)	153 (17.3%)
	50 – 59 years	114 (10.4%)	23 (10.9%)	91 (10.3%)
	60 – 69 years	110 (10.1%)	25 (11.8%)	85 (9.6%)
	≥ 70 years	97 (8.9%)	14 (6.6%)	83 (9.4%)
Sex	Female	535 (48.9%)	99 (46.9%)	436 (49.4)
	Male	559 (51.1)	112 (53.1%)	447 (50.6%)
TB History	No prior TB	1,018 (93.1%)	176 (83.4%)	842 (95.4%)
	First line only	48(4.3%)	23 (10.9%)	25 (2.8%)
	Second line	28 (2.6%)	12 (5.7%)	16 (1.8%)
HIV	Negative	404 (36.9%)	9 (4.3%)	395 (44.7%)
	Positive	690 (63.1%)	202 (5.7%)	488 (55.3%)
CD4 count	< 50 cells/mL	58 (8.4%)	20 (9.9%)	38 (7.8%)
	50 – 99 cells/mL	100 (14.5%)	28 (13.9%)	72 (14.8%)
	100 – 249 cells/mL	170 (24.6%)	51 (25.2%)	119 (24.4%)
	250 – 349 cells/mL	203 (29.4%)	53 (26.2%)	150 (30.7%)
	350 – 499 cells/mL	73 (10.6%)	22 (10.9%)	51 (10.5%)
	≥ 500	86 (12.5%)	28 (13.9%)	58 (11.9%)
ART before admission		217 (31.4%)	54 (26.7%)	163 (33.4%)
ART during admission		255 (37%)	78 (38.6%)	177(6.2%)

**Table 2:** Radiologic characteristics of inpatients with diagnosis of pulmonary tuberculosis admitted to the medical wards of a tertiary, referral hospital in Sub-Saharan Africa

		**Cohorted**		**General**	
Diagnosis of pneumonia (fever + pulmonary infiltrates of any aetiology)		211		132	
Radiological severity	No X ray available	9	(4%)	24	(18%)
	Normal	13	(6%)	13	(10%)
	Mild	75	(36%)	28	(21%)
	Moderate	65	(31%)	33	(25%)
	Severe	49	(23%)	34	(26%)
Cavitary lesions	Not cavitary	161	(76%)	121	(92%)
	Cavitary	50	(24%)	11	(8%)
Radiological extension	Unilateral	127	(60%)	55	(42%)
	Bilateral	84	(40%)	77	(58%)

**Table 3:** Inpatient and one-year TB rates and mortality of patients admitted to the medical wards of a tertiary, referral hospital in Sub-Saharan Africa

Sub-population*	**No. patients**	**No. of days admitted**	**No. of days in cohorting**	**No. of days with an index case in the same bay**	**No. of days adjacent to index case**	**Average TB exposure index (SD)**	**Overall inpatient mortality (%)**	**TB incidence per 100 persons per year of follow up**	**Overall Mortality per year of follow-up (%)**	**TB-related mortality per year of follow up (%)**
**Place of admission**										
General bay only	883	5475	NA	1035	475	6.6 (5.9)	168 (19.0)	16	17	10
General bay AND cohorting bay	80	936	504	1113	432	18.8 (8.6)	2 (2.5)	10	9	6
Cohorting bay only	131	2319	1349	3211	1576	33.2 (16.1)	8 (6.1)	15	31	6
**HIV status**										
Positive	690	5728	1795	3894	1787	12. (14.1)	118 (17.1)	37	43	21
Negative	404	3001	58	1465	696	8.0 (6.3)	60 (14.9)	4	14	1
**CD4 cell count**										
< 50 cells/mL	58	435	198	598	205	11.2 (11.5)	4 (6.9)	2	2	4
50 – 99 cells/mL	100	880	272	625	283	12.8 (14.0)	14 (14)	6	3	4
100 - 199 cells/mL	170	1343	448	613	434	12.0 (13.7)	26 (15.3)	6	4	10
200 – 349 cells/mL	203	1786	440	759	393	11.1 (12.8)	35 (17.3)	9	5	9
350 – 499 cells/mL	73	664	183	672	209	13.2 (15.1)	15 (20.5)	7	5	9
>500 cells/mL	86	619	255	627	263	8.0 (6.3)	24 (28.0)	7	2	7
**Diagnosis of PTB during admission**										
PTB	215	3139	1803	2876	1978	27.1 (15.8)	11 (5.1)	28	17	26
No TB	979	5590	50	2483	505	6.6 (5.9)	167 (17.1)	13	5	31

*The analysis does not include medical patients that died at the Emergency Department, before arriving to the medical wards.

The number of days spent in the same bay with an index case also include the number of days spent with a TB index case adjacent to the patient’s bed.

### During the follow up period

77/896 (8.6%) patients were considered lost-to-follow up (or untraceable). During the 1-year follow up period, 41/896 patients developed confirmed PTB. The overall (all-cause) one-year mortality during follow up was 14% (123/896 patients). Of these, 19/123 (15%) had microbiologically proven TB and 7/123 (6%) had clinically diagnosed TB (Table 3). 72/896 patients were initially admitted to the general medical ward without a suspicion for PTB (Additional file 2).

### Factors associated with active TB and TB-associated mortality post-discharge

One-year TB incidence and one-year TB-associated mortality were associated with admission in the cohorting bay, HIV infection, CD4 < 100 cells/mL, and PTB diagnosis during admission (Table 3). Bivariate analysis is shown in the Additional file 3. In multivariable analysis, one-year TB incidence was independently associated with the number of days that the patient remained hospitalized and proximity to TB index cases (exposure index) among patients without HIV infection (Table 4). Among HIV-infected patients, one-year TB incidence was independently associated with the number of days that the patient remained hospitalized, but the association with the exposure index was only found among HIV-infected patients who did not have a PTB diagnosis during admission. As expected, history of prior TB infection (particularly resistant TB), a diagnosis of PTB during hospitalization and older age were also associated with TB incidence during follow up (Table 4).

**Table 4:** Factors associated with development of pulmonary tuberculosis within 12 months after discharge from a tertiary hospital in Botswana

		**Models including participants who were diagnosed with TB during their inpatient admission, n = 916**				**Models excluding participants who were diagnosed with TB during their inpatient admission, n = 713**			
**Variable**		**AOR (95% CI)**	**AOR (95% CI)**	**AOR (95% CI)**	**AOR (95% CI)**	**AOR 95% C I**	**AOR (95% CI)**	**AOR (95% CI)**	**AOR (95% CI)**
**Age**	**21 - 40 years**	Reference	Reference	Reference	Reference	Reference	Reference	Reference	Reference
	**41 - 60 years**	1.3 (0.4-4.9)	0.9 (0.3-5.4)	2.5 (0.6-10.7)	2.5 (0.6-10.8)	1.1 (0.2-6.2)	0.9 (0.2-5.8)	1.2 (0.2-8.4)	1.2 (0.2-8.4)
	**> 60 years**	0.9 (0.2-3.5)	1.1 (0.3-4.0)	1.3 (0.2-7.8)	1.5 (0.3-7.6)	2.2 (0.4-10.6)	2.1 (0.4-10.8)	2.9 (0.4-20.5)	2.6 (0.5-14.3)
**Sex (male)**		1.5 (0.5-4.2)	1.3 (0.5-3.5)	1.7 (0.6-5.1)	1.5 (0.5-4.4)	0.9 (0.2-3.6)	0.9 (0.2-3.6)	0.9 (0.2-4.2)	0.9 (0.2-3.9)
HIV infection*		3.9 (0.8-20.5)	2.9 (0.6-13.1)	2.7 (0.5-15.5)	3.6 (0.6-20.0)	2.8 (0.4-18.4)	2.7 (0.4-17.6)	1.6 (0.2-12.6)	1.6 (0.2-11.7)
CD4 cell* count	**CD4 > 350**	Reference	Reference	Reference	Reference	Reference	Reference	Reference	Reference
	**CD4 100 - 350**	0.9 (0.4-2.1)	0.9 (0.3-2.0)	0.8 (0.2-2.2)	0.9 (0.4-2.1)	0.8 (0.4-2.0)	1.2 (0.3-8.6)	2.2 (0.4-10.6)	3.4 (0.4-28.8)
	**CD4 < 100**	3.1 (1.3-7.5)	3.6 (1.1–6.9)	3.2 (1.2-7.2)	2.9 (1.2-7.1)	2.8 (1.1-6.6)	3.1 (1.1-12.6)	3.0 (1.1-18.4)	6.5 (0.7-61.3)
**Prior history of TB treatment**		12.4 (3.7-41.1)	11.9 (3.8-37.4)	4.6 (1.2-17.4)	6.8 (2.0-23.6)	10.6 (2.6-44.0)	7.8 (1.7-36.0)	2.4 (0.4-16-4)	3.1 (0.5-18.6)
**Total days admitted to the hospital**		2.0 (1.1-3.6)	NA	NA	NA	1.4 (0.6-3.2)	NA	NA	NA
**Days spent in the same bay with a TB index case**		NA	1.8 (1.1-2.9)	NA	NA	NA	1.5 (0.4-4.4)	NA	NA
**Days of adjacent exposure to a TB index case**		NA	NA	4.6 (2.2-9.9)	NA	NA	NA	3.4 (1.3-8.9)	NA
**TB exposure index**		NA	NA	NA	1.9 (1.3-2.8)	NA	NA	NA	2.4 (1.3-4.2)
**Inpatient diagnosis of TB**		2.6 (0.8-8.6)	1.6 (0.5-5.1)	1.9 (0.6 -6.0)	1.8 (0.6-5.6)				

*Given collinearity, models including HIV and CD4 cells were developed separately.

Similarly, different models were developed for the variables: a) total days admitted to the hospital; b) days spent in the same bay with a TB index case; c) days of adjacent to a TB index case; and, d) TB exposure score.

Total days admitted to the hospital, days spent in the same bay with a TB index case, days of adjacent to a TB index case were modelled as increments by 10 (days). TB exposure index was modelled as an ordinal variable with increments by 1 point. To calculate the daily exposure index per participant per day, adjacent cases were given twice the weight of non-adjacent ones. For example, if a participant had 2 adjacent TB cases and one non-adjacent TB patient in his/her bay, the “exposure index” for that day would be calculated as: 2x2+1=5.

## Discussion

In this study we document the very high rates of nosocomial TB exposure and high levels of one-year TB incidence and mortality after discharge from a tertiary hospital in Sub-Saharan Africa. Our study identified four important groups of patients that may deserve special attention and, perhaps, targeted interventions given their very high rates of nosocomial TB exposure, one-year TB incidence and one-year mortality: a) patients with long hospitalization periods; b) patients cohorted in the TB bay (regardless of their diagnosis of TB); c) patients diagnosed with TB during their hospitalization; and d) HIV-infected patients with severe immunosuppression. Due to their potentially high risk for development of active TB, education regarding the signs and symptoms of TB at discharge and close follow up may be recommended. Given the high levels of documented exposure, further preventive interventions such as active follow-up of these patients and/or isoniazid prophylactic therapy may be useful [35-37]. Studies to determine the role of such interventions are needed.

Contrary to what we expected, we found that the total number of days of hospitalization, the total number of days spent at the cohorting bay, the proximity and number of TB index cases that contribute to the cumulative nosocomial TB exposure and the burden of the index TB case disease (quantified by AFB microscopy) had similar levels of association for incident TB. As expected, however, we found that the relationship between cumulative TB exposure and one-year TB incidence was different by HIV status. However, although the trends and values were different, the probability to develop incident TB after discharge from the hospital increased as the cumulative TB exposure increased, without reaching a plateau. These curves are not comparable to each other but they provide valuable information regarding the effect, type and patterns in which exposure to TB is associated with TB incidence (data not shown).

Not unexpectedly, we document significant differences in TB incidence by cumulative exposure to TB patients cohorted on the TB bay. Although the lack of control group (hospitals that do not cohort TB patients) does not allows us to draw conclusions about the efficacy of this strategy in decreasing the overall net nosocomial TB exposure, it does highlights the nature of the resulting exposure and identify potential future targets for further intervention. Point-of-care diagnostic tools for TB, such as the recently developed GeneXpert MTB/RIF are likely to contribute significantly to the accurate triage of patients on admission [15,16,38,39]. Such triage will likely decrease TB exposure if other infection control measures are also maximized. However, given the high prevalence of other factors leading to nosocomial TB exposure in busy hospitals where TB and HIV infection are highly prevalent, the impact of such technologies in reducing nosocomial TB transmission remains to be determined.

Our study has several limitations. First, in spite of all our attempts to quantify the variables that could potentially lead to nosocomial TB transmission, there are no gold-standards to quantify the degree of exposure. Our surrogates (distance between patients and position in the bay), can only give us a rough idea of the magnitude of the level of exposure and risk for TB acquisition at the hospital. Furthermore, the scale used to compare the degree of exposure between the groups may fail to include other factors that are likely to influence the risk for nosocomial TB transmission such as host immunosuppression and immune-related factors, virulence of the pathogen, infectiousness of the index case and environmental factors among others. We believe that the development of more accurate measures of exposure will significantly improve the study of TB transmission in the future and research in that area needs to be prioritized. Nevertheless, in spite of its limitations, our attempt to measure the intensity of exposure was useful to demonstrate a significant difference in the exposure of patients admitted to the general ward when compared to the patients admitted to the TB cohorting bay. Second, our study was conducted over a 3-month period and 1 years of follow-up, providing only a snapshot of the situation. The lack of active case finding through sputum induction and culture of patients with cough, likely led to an underestimation of the number of active TB cases admitted to the wards during that time period. Similarly, the lack of a prospective follow up of the patients admitted to the wards during the study period does not allow us to estimate the impact of such exposure on transmission of disease. Our score would need to be correlated with the rate of development of TB infection among a closely-monitored cohort of patients in order to make it clinically meaningful. Fully aware of these limitations, we do not recommend the use of our scoring system used in our study to be used for any clinical or infection control decisions. As mentioned, the main objective of the scoring system was to allow the comparison between different settings in the medical ward.

In spite of these limitations, we believe the results of our study accurately depict the situation at a major hospital in a high HIV and TB prevalence area under real-world implementation conditions. Given that the environmental conditions, patient population and hospital procedures are similar to the ones present at a large number of high-volume large referral centres in areas with high prevalence of HIV and TB infection, we believe our results are also generalizable to similar settings. Our study highlights some of the difficulties for the implementation of effective infection control measures aims to reduce TB exposure among inpatients in low-resource settings. Until the best way to reduce such exposure is determined, simple but effective infection control measures that may decrease TB exposure must be prioritized. Among others, outpatient management of TB patients should be encouraged when possible. Rapid diagnosis, initiation of treatment and discharge should be implemented for patients admitted to the hospital. Appropriate cross-ventilation, as well as the use of surgical masks and cough hygiene practices on patients known to have TB should also be performed. Studies to determine the best way to triage patients being admitted to the hospital and minimize the TB exposure of patients at highest risk for nosocomial TB acquisition are in high need.

## Conclusion

The number of days spent in medical wards in Botswana is strongly associated with development of TB within the following 12 months after discharge. TB-related mortality is very high among these patients. TB incidence and TB-associated mortality during follow up are associated with the overall time spent as an inpatient, the time spent at the cohorting bay, the time spent contiguously to a TB index case.

## Competing interests

The authors declare no competing interests.

## Authors’ contributions

Dr NZ had full access to all the data in the study and take responsibility for the integrity of the data and the accuracy of the data analysis. Study concept and design: CM, NZ, NM, AP. Acquisition of data: CM, NZ, NM, RC, RN, NM, AP. Analysis and interpretation of data: CM, NZ, RN, KJ, RC, SS. Drafting of manuscript: NZ, NM. Critical revision of the manuscript for important intellectual content: CM, NZ, RN, SS, JK, RC. Statistical analysis: CM, NZ, NM, AP. Obtaining funding: NZ. Administrative, technical, or material support: CM, AP, NM. Study supervision: NZ, RC. All authors read and approved the final manuscript.

## Pre-publication history

The pre-publication history for this paper can be accessed here:

http://www.biomedcentral.com/1471-2334/14/409/prepub

## Supplementary Material

Additional file 1: Figure S1 Decision algorithm for patient triage to a tubeculosis cohorting bay. Click here for file

Additional file 2 Shows the distribution of patients according to their suspicion for tuberculosis on admission, allocation on the ward and ultimate diagnosis of pulmonary tuberculosis. Click here for file

Additional file 3: Table S1 Bivariate analysis of the main co-factors potentially associated with one-year incident tuberculosis and one-year mortality. Click here for file
