# Erratum: Longer hospital stay is associated with high rates of tuberculosis-related morbidity and mortality within 12 months after discharge in a referral hospital in Sub-Saharan Africa

**DOI:** 10.1186/s12879-015-0942-8

**Published:** 2015-05-10

**Authors:** Nicola M Zetola, Nenad Macesic, Sanghyuk S Shin, Alexandra Peloso, Ronald Ncube, Jeffrey D Klausner, Chawangwa Modongo, Ronald G Collman

**Affiliations:** Division of Infectious Disease, University of Pennsylvania, Philadelphia, PA USA; University of Botswana Medical School, University of Botswana, Gaborone, Botswana; Botswana-UPenn Partnership, Gaborone, Botswana; Division of Infectious Diseases, University of Melbourne, Melbourne, Australia; School of Medicine, University of California, Los Angeles, USA; University of Western Ontario, School of Medicine, London, Canada; Botswana National TB Program, Gaborone, Botswana; Division of Pulmonary and Critical Care Medicine, University of Pennsylvania, Philadelphia, PA USA

## Erratum

Following publication of the original article [1] we became aware of the following errors which we wish to correct. These corrections have no impact over the study results, their interpretation or conclusions.

### Authors and affiliations

The correct list of authors and their respective affiliations is the following:

Nicola M Zetola^1,2,3^, Nenad Macesic^4^, Sanghyuk S. Shin^5^**, Alexandra Peloso**^**6**^, Ronald Ncube^7^, **Jeffrey D Klausner**^**5**^, Chawangwa Modongo^1,3^, Ronald G Collman^8^

1. Division of Infectious Disease, University of Pennsylvania, Philadelphia, Pennsylvania;
2. University of Botswana Medical School, University of Botswana, Gaborone, Botswana;
3. Botswana-UPenn Partnership, Gaborone, Botswana;
4. Division of Infectious Diseases, University of Melbourne, Melbourne, Australia;
5. University of California, Los Angeles, Los Angeles, California;
6. University of Western Ontario; School of Medicine, Botswana,
7. Botswana National TB Program, Gaborone, Botswana
8. Division of Pulmonary and Critical Care Medicine, University of Pennsylvania, Philadelphia, Pennsylvania

### Abstract

1. The following text “Eventually, 916 (83.7%) patients were discharged and followed for one year after it. Of these, 51 (5.6%) were diagnosed with PTB during the year of follow up (annual TB rate of 3,712 cases per 100,000 person per year)” should be replaced with **"Of the 896 (83.7%) discharged patients, 41 (4.6%) were diagnosed with TB during the year of follow-up. Overall, 123/896 (14%) patients died during the follow up period, of whom 26/123 (21%) died from TB.”**

### Table 3

2. The row ‘No TB’ (last row [row 18]) should read **879**, not 979.
3. The column ‘No. of Patients’ (column 2) should also read **879**, not 979.
4. The column entitled “TB incidence per 100 persons per year of follow up” (column 9) should be entitled **“TB incidence during the one-year follow up”**.
5. There is a typo in the column “Average TB exposure index” (column 7), row “HIV status positive” (row 7). The decimal number is missing. It reads ”12.” and should be **“12.3”**.
6. In the last 2 columns, the frequencies under the subheading “**CD4 cell count**” and “**Diagnosis of PTB during admission**” have been switched. That is, the numbers currently under the “TB-related mortality” column (the last column; n = 4, 4, 10, 9, 9, 7) belong to the “Overall Mortality” column (second to last column; n = 2, 3, 4, 5, 5, 2) and vice versa.
7. Same as above, the numbers for “**Diagnosis of TB at admission**” (n = 17, 5) should be in the last column (“TB-related mortality”) and the numbers 26, 31 should be in the “Overall Mortality” (second to last) column.
8. The table headings of the last 2 columns should reflect that these are **(n)’s,** not (%)’s.

### Table 4

9. The sample size for the column entitled “Models including participants who were diagnosed with TB during their inpatient admission” is **896**, not 916.

### In additional file 3

10 . The sample size should be noted as **n = 896**.
11 . The column heading “Incident TB during 1-year follow-up” and “Death during 1-year of follow-up” should indicate that the reported measures are **unadjusted odds ratios**.
