# Supplementary material for: Longer hospital stay is associated with higher rates of tuberculosis-related morbidity and mortality within 12 months after discharge in a referral hospital in Sub-Saharan Africa
Source: BMC Infect Dis. 2014 Jul 22;14:409. doi: 10.1186/1471-2334-14-409 (PMC4223402; doi:10.1186/1471-2334-14-409)

**INFECTION CONTROL MEASSURES IN PLACE:**

**Specific facility-level activities – managerial**

**Implement the set of facility-level managerial activities.** Facility-level managerial activities include identification and strengthening of local coordinating bodies and development of a facility plan (including human resources) for implementation of TB infection control. The hospital’s plan includes policies and procedures to ensure proper implementation of the administrative controls, environmental controls and use of particulate respirators. On-site surveillance of TB disease among health workers and assessment of facility is routinely performed. Monitoring and evaluation, and participation in research efforts, are also in line with the national research agenda.

**Specific facility-level controls – administrative**

These measures include promptly identify people with TB symptoms (triage), separate infectious patients, control the spread of pathogens (cough etiquette and respiratory hygiene) and minimize time spent in health-care facilities

- **Promptly identify people with TB symptoms (triage).** Prompt identification of people with TB symptoms (i.e. triage) is crucial. The specific criteria for triaging patients and algorithm used at our facility are detailed in the figure below. In general, people suspected of having TB is separated from other patients, cohorted in ventilated areas, educated on cough etiquette and respiratory hygiene. In particular, the following triage algorithm was used at the hospital were this study was conducted:


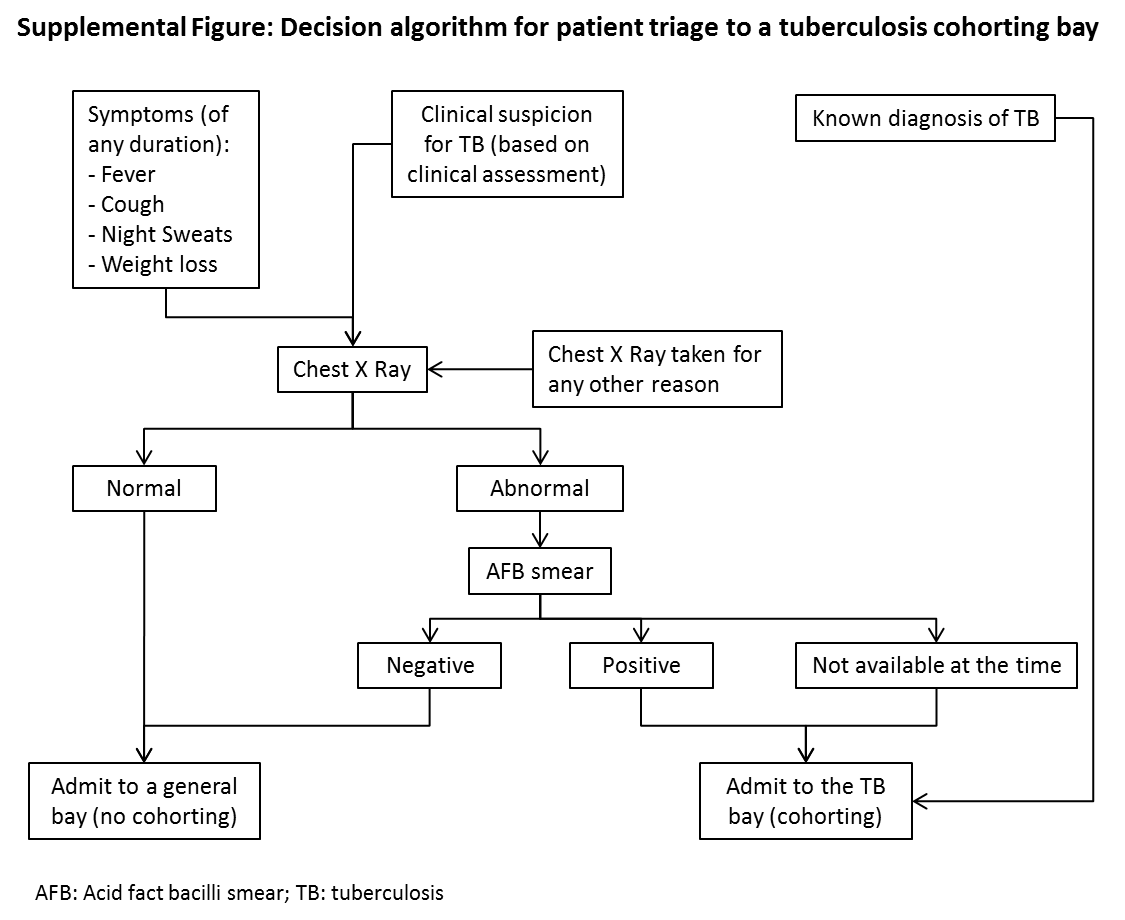

Supplement: Additional file 1: Figure S1 — Decision algorithm for patient triage to a tubeculosis cohorting bay. [file 1471-2334-14-409-S1.docx]
