# Supplementary material for: Longer hospital stay is associated with higher rates of tuberculosis-related morbidity and mortality within 12 months after discharge in a referral hospital in Sub-Saharan Africa
Source: BMC Infect Dis. 2014 Jul 22;14:409. doi: 10.1186/1471-2334-14-409 (PMC4223402; doi:10.1186/1471-2334-14-409)
Supplement: Additional file 2 — Shows the distribution of patients according to their suspicion for tuberculosis on admission, allocation on the ward and ultimate diagnosis of pulmonary tuberculosis. [file 1471-2334-14-409-S2.pptx]

## Slide 1
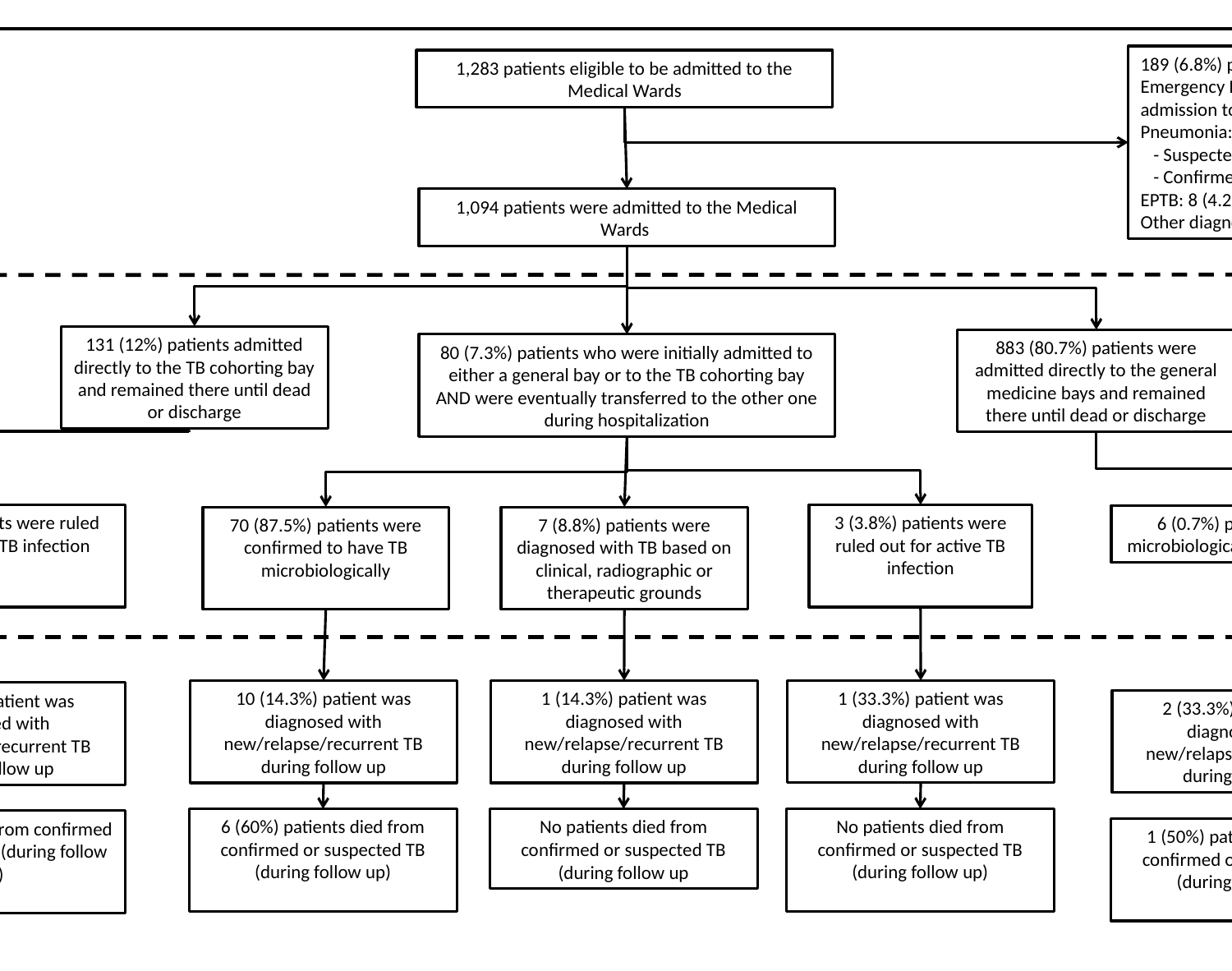

189 (6.8%) patients died at the Emergency Department before admission to the medical wards:
Pneumonia: 34 (18%)
 - Suspected TB: 16
 - Confirmed TB: 4
EPTB: 8 (4.2%)
Other diagnosis 147 (77.8%)
1,283 patients eligible to be admitted to the Medical Wards
1,094 patients were admitted to the Medical Wards
Before admission:
189 (6.8%) patients died before admission (in the Emergency Department)
Of them, 20 (10.6%) died form suspected or confirmed TB
During hospitalization:
12 (5.6%) of the 215 patients who were diagnosed with TB during their admission died before discharge. Considering that 178 (16.3%) of the 1,094 inpatients died before discharge, 6.7% (12 of 178) of the inpatient period mortality was attributable to TB. 14 (7.9%) additional patients died with pneumonia of undetermined aetiology.
131 (12%) patients admitted directly to the TB cohorting bay and remained there until dead or discharge
883 (80.7%) patients were admitted directly to the general medicine bays and remained there until dead or discharge
80 (7.3%) patients who were initially admitted to either a general bay or to the TB cohorting bay AND were eventually transferred to the other one during hospitalization
115 (87.8%) patients were confirmed to have TB microbiologically
13 (9.9%) patients were diagnosed with TB based on clinical, radiographic or therapeutic grounds
3 (2.3%) patients were ruled out for active TB infection
3 (3.8%) patients were ruled out for active TB infection
6 (0.7%) patients had a microbiological diagnosis of TB
 4 (0.5) patients were clinically diagnosed with TB and started on treatment
873 (98.8%) never presented clinical evidence of TB or were ruled out for TB infection
70 (87.5%) patients were confirmed to have TB microbiologically
7 (8.8%) patients were diagnosed with TB based on clinical, radiographic or therapeutic grounds
During follow-up: Of the 916 patients who were eventually discharged for the Medical Wards, 51 (5.6%) developed TB during the first year of follow up. Of them 26 (51%) died.
1 (33.3%) patient was diagnosed with new/relapse/recurrent TB during follow up
No patients died from confirmed or suspected TB (during follow up)
10 (14.3%) patient was diagnosed with new/relapse/recurrent TB during follow up
6 (60%) patients died from confirmed or suspected TB (during follow up)
1 (14.3%) patient was diagnosed with new/relapse/recurrent TB during follow up
No patients died from confirmed or suspected TB (during follow up
1 (33.3%) patient was diagnosed with new/relapse/recurrent TB during follow up
No patient died from confirmed or suspected TB (during follow up)
15 (13.1%) patients were diagnosed with new/relapse/recurrent TB during follow up
10 (66.7%) patients died from confirmed or suspected TB (during follow up)
4 (30.8%) patients were diagnosed with new/relapse/recurrent TB during follow up
3 (75%) patients died from confirmed or suspected TB (during follow up)
 16 (18.3%) patient was diagnosed with new/relapse/recurrent TB during follow up
6 (37.5%) patients died from confirmed or suspected TB (during follow up)
2 (33.3%) patient was diagnosed with new/relapse/recurrent TB during follow up
1 (50%) patient died from confirmed or suspected TB (during follow up)
 1 (25%) patient was diagnosed with new/relapse/recurrent TB during follow up
No patients died from confirmed or suspected TB (during follow up)

## Slide 2
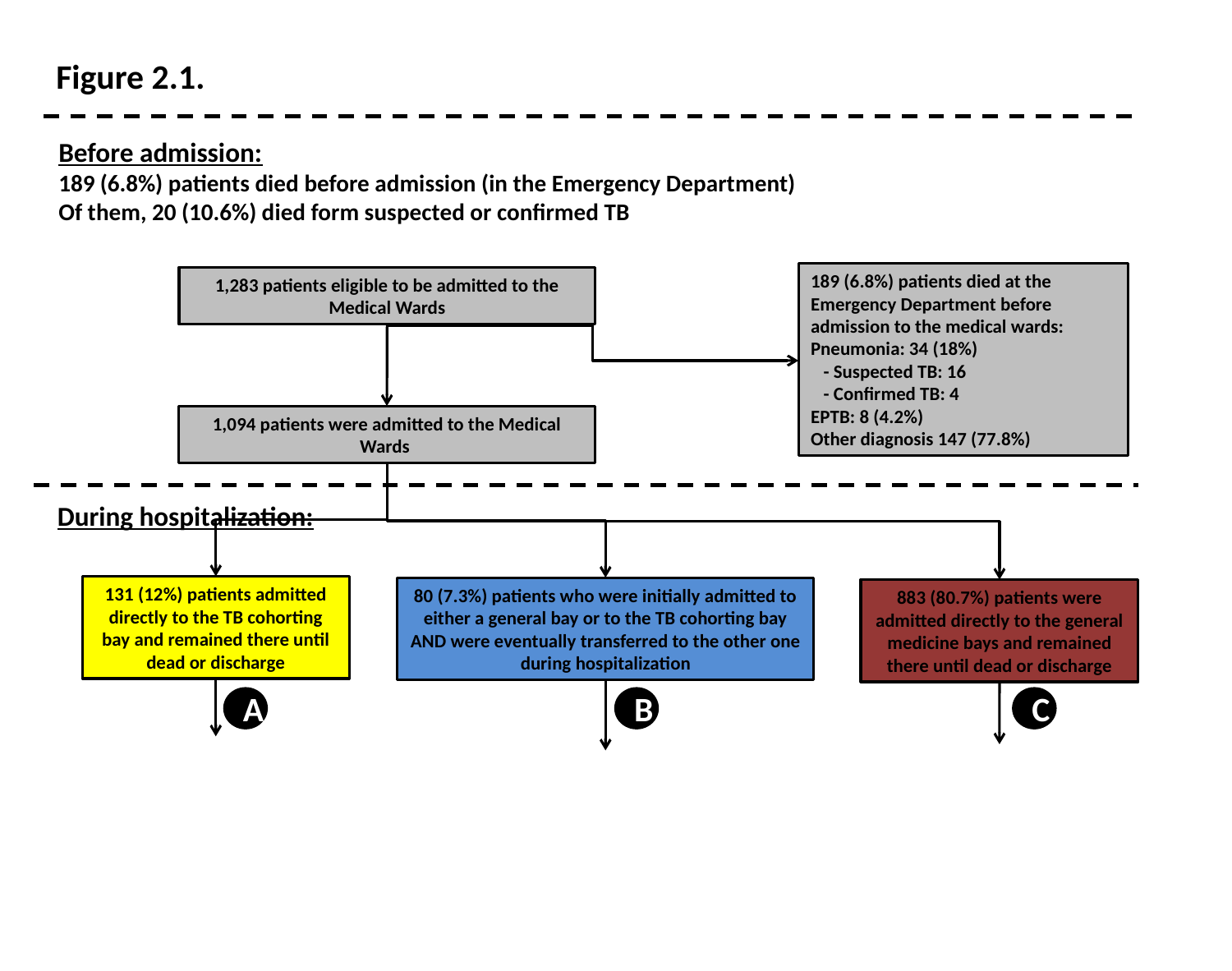

Figure 2.1.
Before admission:
189 (6.8%) patients died before admission (in the Emergency Department)
Of them, 20 (10.6%) died form suspected or confirmed TB
189 (6.8%) patients died at the Emergency Department before admission to the medical wards:
Pneumonia: 34 (18%)
 - Suspected TB: 16
 - Confirmed TB: 4
EPTB: 8 (4.2%)
Other diagnosis 147 (77.8%)
1,283 patients eligible to be admitted to the Medical Wards
1,094 patients were admitted to the Medical Wards
During hospitalization:
131 (12%) patients admitted directly to the TB cohorting bay and remained there until dead or discharge
80 (7.3%) patients who were initially admitted to either a general bay or to the TB cohorting bay AND were eventually transferred to the other one during hospitalization
883 (80.7%) patients were admitted directly to the general medicine bays and remained there until dead or discharge
A
B
C

## Slide 3
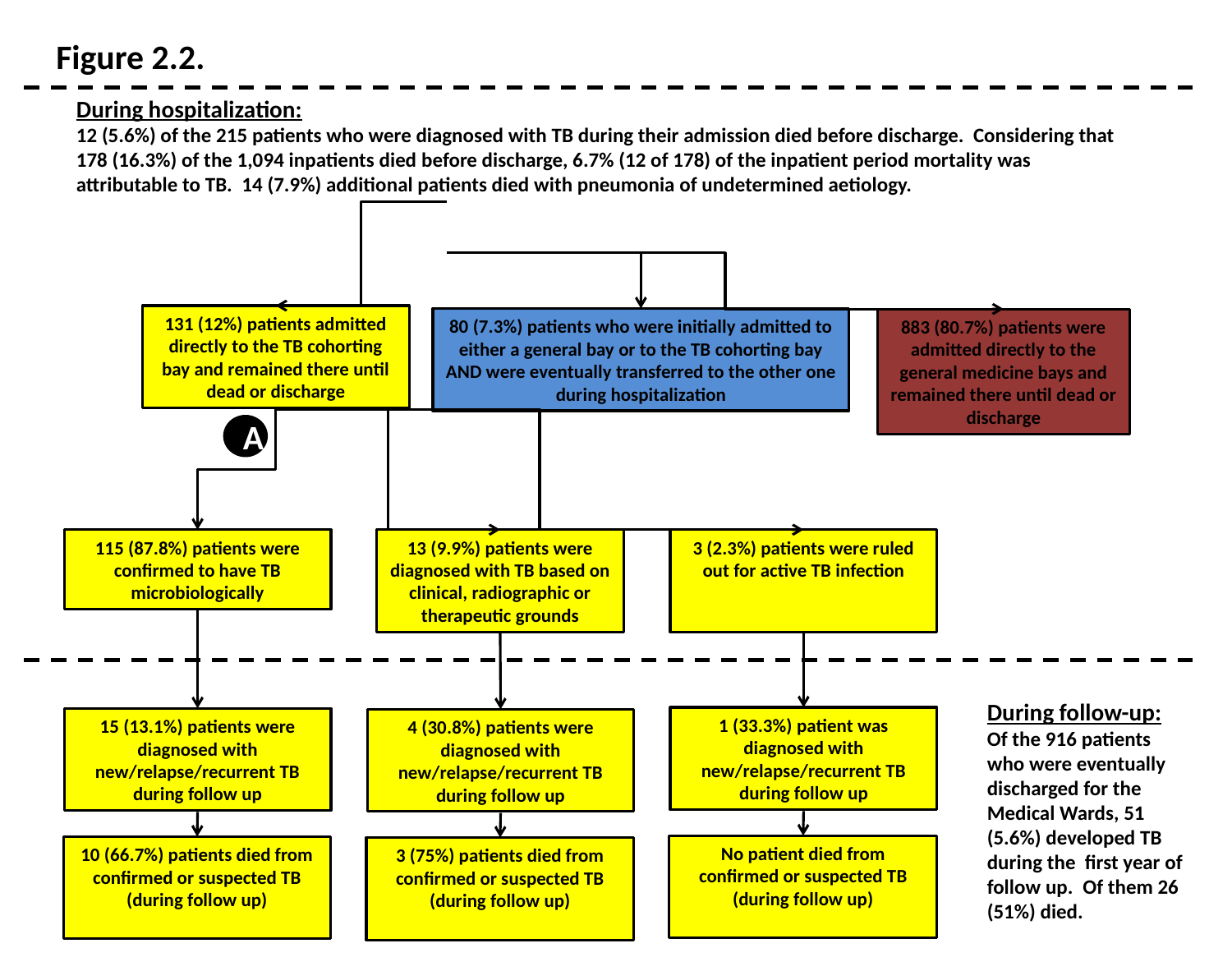

Figure 2.2.
During hospitalization:
12 (5.6%) of the 215 patients who were diagnosed with TB during their admission died before discharge. Considering that 178 (16.3%) of the 1,094 inpatients died before discharge, 6.7% (12 of 178) of the inpatient period mortality was attributable to TB. 14 (7.9%) additional patients died with pneumonia of undetermined aetiology.
131 (12%) patients admitted directly to the TB cohorting bay and remained there until dead or discharge
80 (7.3%) patients who were initially admitted to either a general bay or to the TB cohorting bay AND were eventually transferred to the other one during hospitalization
883 (80.7%) patients were admitted directly to the general medicine bays and remained there until dead or discharge
A
115 (87.8%) patients were confirmed to have TB microbiologically
13 (9.9%) patients were diagnosed with TB based on clinical, radiographic or therapeutic grounds
3 (2.3%) patients were ruled out for active TB infection
During follow-up: Of the 916 patients who were eventually discharged for the Medical Wards, 51 (5.6%) developed TB during the first year of follow up. Of them 26 (51%) died.
1 (33.3%) patient was diagnosed with new/relapse/recurrent TB during follow up
No patient died from confirmed or suspected TB (during follow up)
15 (13.1%) patients were diagnosed with new/relapse/recurrent TB during follow up
10 (66.7%) patients died from confirmed or suspected TB (during follow up)
4 (30.8%) patients were diagnosed with new/relapse/recurrent TB during follow up
3 (75%) patients died from confirmed or suspected TB (during follow up)

## Slide 4
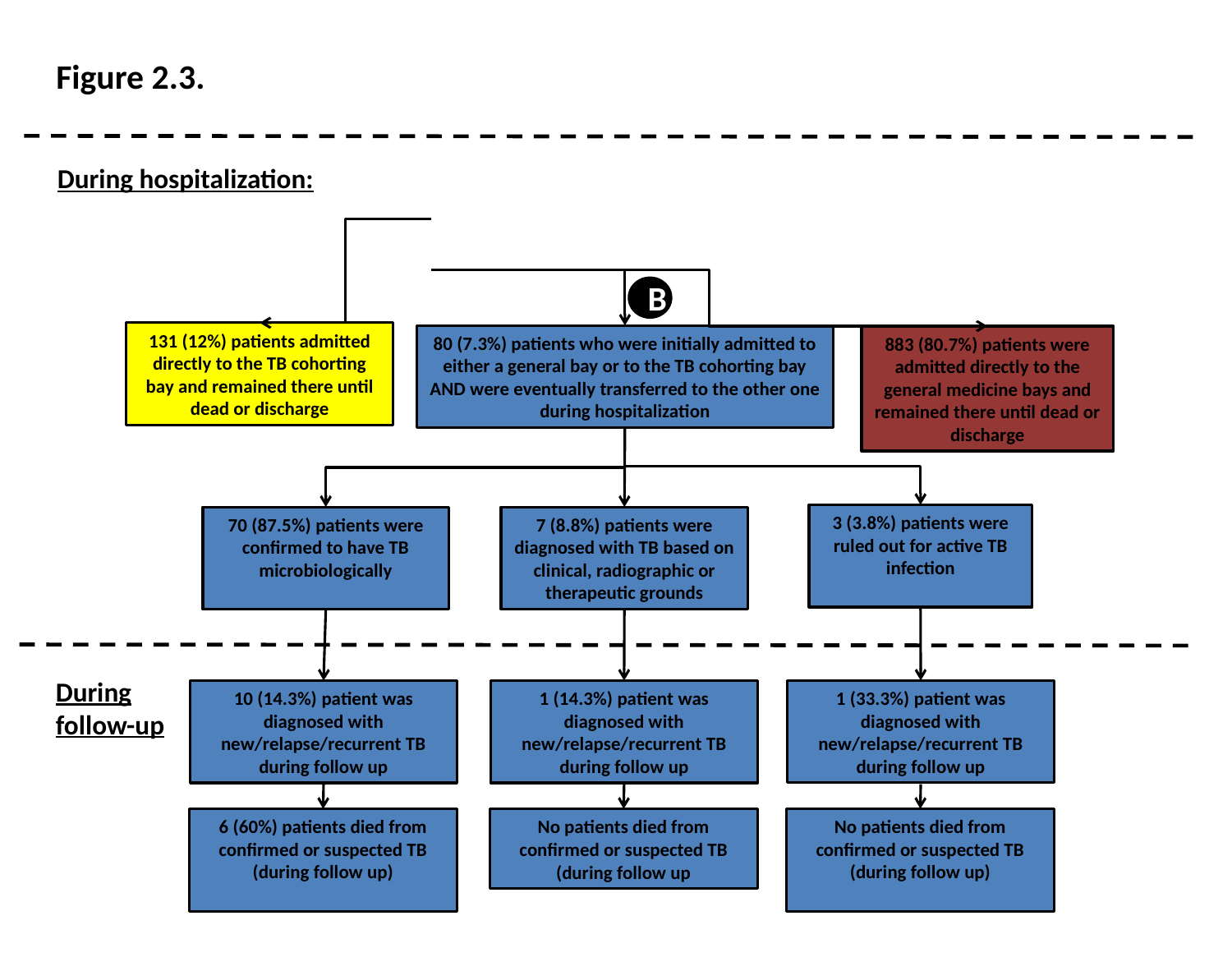

Figure 2.3.
During hospitalization:
131 (12%) patients admitted directly to the TB cohorting bay and remained there until dead or discharge
80 (7.3%) patients who were initially admitted to either a general bay or to the TB cohorting bay AND were eventually transferred to the other one during hospitalization
883 (80.7%) patients were admitted directly to the general medicine bays and remained there until dead or discharge
B
3 (3.8%) patients were ruled out for active TB infection
70 (87.5%) patients were confirmed to have TB microbiologically
7 (8.8%) patients were diagnosed with TB based on clinical, radiographic or therapeutic grounds
During follow-up
1 (33.3%) patient was diagnosed with new/relapse/recurrent TB during follow up
No patients died from confirmed or suspected TB (during follow up)
10 (14.3%) patient was diagnosed with new/relapse/recurrent TB during follow up
6 (60%) patients died from confirmed or suspected TB (during follow up)
1 (14.3%) patient was diagnosed with new/relapse/recurrent TB during follow up
No patients died from confirmed or suspected TB (during follow up

## Slide 5
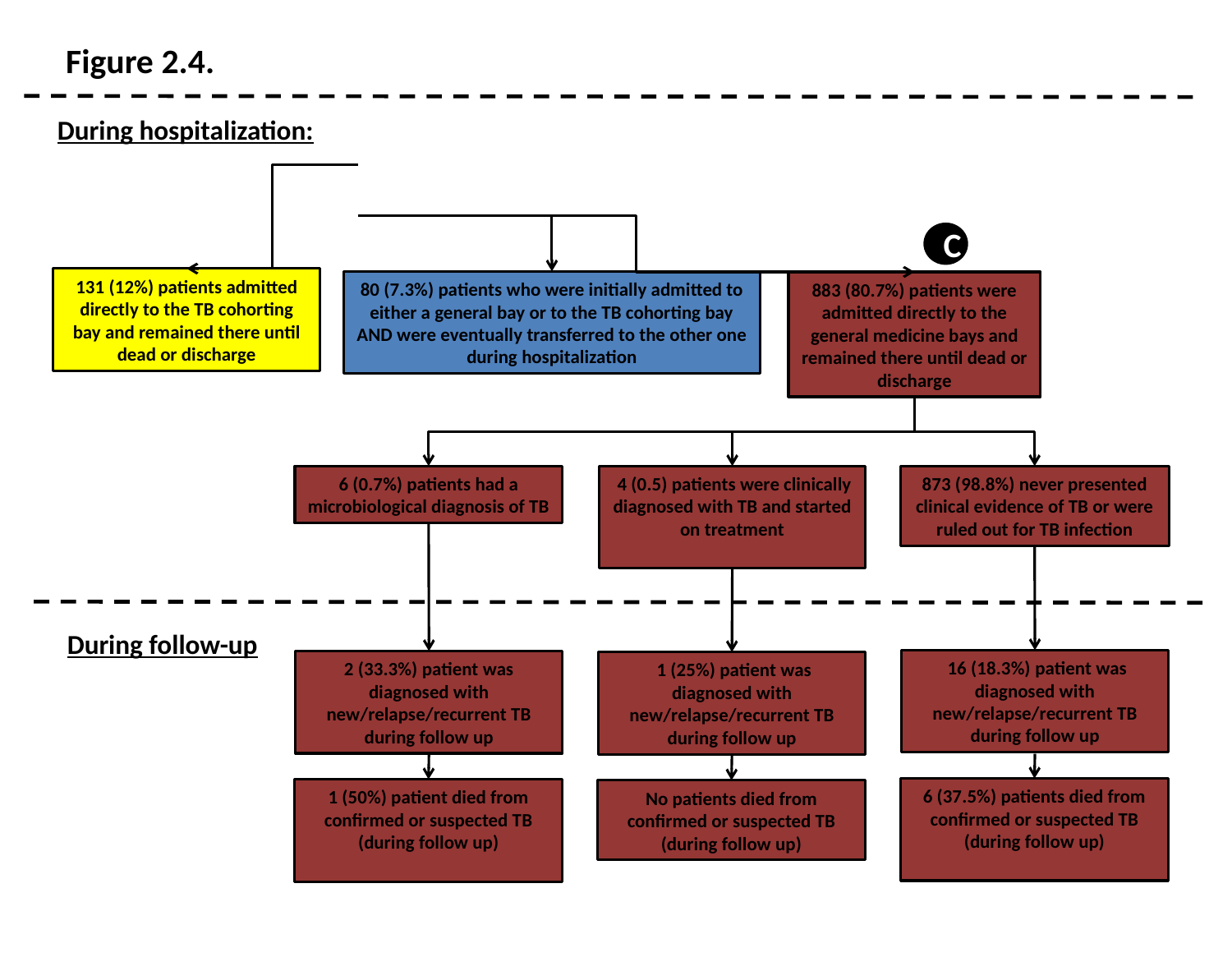

Figure 2.4.
During hospitalization:
131 (12%) patients admitted directly to the TB cohorting bay and remained there until dead or discharge
80 (7.3%) patients who were initially admitted to either a general bay or to the TB cohorting bay AND were eventually transferred to the other one during hospitalization
883 (80.7%) patients were admitted directly to the general medicine bays and remained there until dead or discharge
C
6 (0.7%) patients had a microbiological diagnosis of TB
 4 (0.5) patients were clinically diagnosed with TB and started on treatment
873 (98.8%) never presented clinical evidence of TB or were ruled out for TB infection
During follow-up
 16 (18.3%) patient was diagnosed with new/relapse/recurrent TB during follow up
6 (37.5%) patients died from confirmed or suspected TB (during follow up)
2 (33.3%) patient was diagnosed with new/relapse/recurrent TB during follow up
1 (50%) patient died from confirmed or suspected TB (during follow up)
 1 (25%) patient was diagnosed with new/relapse/recurrent TB during follow up
No patients died from confirmed or suspected TB (during follow up)
