# Supplementary material for: Longer hospital stay is associated with higher rates of tuberculosis-related morbidity and mortality within 12 months after discharge in a referral hospital in Sub-Saharan Africa
Source: BMC Infect Dis. 2014 Jul 22;14:409. doi: 10.1186/1471-2334-14-409 (PMC4223402; doi:10.1186/1471-2334-14-409)
Supplement: Additional file 3: Table S1 — Bivariate analysis of the main co-factors potentially associated with one-year incident tuberculosis and one-year mortality. [file 1471-2334-14-409-S3.docx]

**Additional file 3: Table S1: Bivariate analysis of the main co-factors potentially associated with one-year incident tuberculosis and one-year mortality.**

| Main Outcomes | | | |  |  |
| --- | --- | --- | --- | --- | --- |
|  | Variable | Incident TB during 1-year follow-up | P value | Death during 1-year of follow-up | P value |
| Age | 18 – 29 years | Reference |  | Reference | - |
|  | 30 – 39 years | 1.23 (0.33 - 4.51) | 0.748 | 1.14 (0.66 - 1.98) | 0.618 |
|  | 40 – 49 years | 0.74 (0.13 - 4.19) | 0.740 | 1.05 (0.55 - 1.99) | 0.869 |
|  | 50 – 59 years | 0.75 (0.13 - 4.20) | 0.740 | 1.06 (0.55 - 2.02) | 0.856 |
|  | 60 – 69 years | 2.47 (0.42 - 14.48) | 0.315 | 1.62 (0.69 - 3.82) | 0.265 |
|  | ≥ 70 years | 0.98 (0.17 - 5.53) | 0.981 | **2.69 (1.47 - 4.92)** | **<0.001** |
| Sex | Male | 1.08 (0.42 - 2.77) | 0.877 | 0.84 (0.59 - 1.21) | 0.377 |
| Prior TB history | No prior TB | Reference | - | Reference | - |
|  | First line only | **8.01 (2.40 - 16.21)** | **<0.001** | 1.01 (0.57 - 1.78) | 0.969 |
|  | Second line | **16.55 (5.91 - 46.37)** | **<0.001** | 4.46 (0.73 - 27.01) | 0.103 |
| Diagnosis of diabetes type 2 | | 1.41 (0.39 - 5.02) | 0.602 | 1.51 (0.92 - 2.45) | 0.096 |
| HIV status | Positive | 2.89 (0.82 - 10.16) | 0.097 | 0.94 (0.65 - 1.37) | 0.777 |
| CD4 cell count* | < 50 cells/mL | 2.06 (0.39 - 10.88) | 0.391 | **2.18 (1.09 - 4.35)** | **0.027** |
|  | 50 – 99 cells/mL | 2.40 (0.57 - 10.08) | 0.232 | 1.29 (0.66 - 2.53) | 0.445 |
|  | 100 – 249 cells/mL | 0.29 (0.03 - 2.86) | 0.289 | **0.45 (0.21 – 0.95)** | **0.038** |
|  | 250 – 349 cells/mL | 0.86 (0.13 - 5.42) | 0.881 | 0.64 (0.30 - 1.37) | 0.255 |
|  | > 350 cells/mL | Reference | - | Reference | - |
| ART before admission* | | 0.58 (0.19 - 1.76) | 0.341 | 0.49 (0.31 - 0.77) | <0.01 |
| ART during admission* | | 0.79 (0.27 - 2.25) | 0.662 | 0.87 (0.54 - 1.39) | 0.573 |
| Diagnosis of pneumonia during admission | | **7.37 (2.10 - 25.88)** | **<0.01** | 1.34 (0.93 - 1.93) | 0.107 |
| Radiologic severity | Normal | Reference |  | Reference |  |
|  | Mild | 1.37 (0.43 – 5.11) | 0.348 | 1.43 (0.30 – 6.89) | 0.740 |
|  | Moderate-severe | 2.47 (0.82 - 10.47) | 0.102 | 3.41 (0.45 – 12.80) | 0.203 |
| Place of admission and hospital stay | General bay only | Reference | - | Reference | - |
|  | General bay AND cohorting bay | **2.42 (1.31 - 10.23)** | **<0.01** | 1.12 (0.71 - 3.13) | 0.128 |
|  | Cohorting bay only | **8.82 (2.51 - 31.01)** | **<0.001** | **1.42 (0.98 - 2.05)** | **0.058** |
| Diagnosis of PTB during admission | | **1.51 (1.08 - 2.10)** | **0.014** | **1.17 (1.01 - 1.35)** | **0.030** |
| Total days admitted to the hospital | | **1.05 (1.01 - 1.12)** | **0.033** | 0.98 (0.96 - 1.01) | 0.085 |
| Days spent adjacent to index case | | **1.21 (1.13 - 1.29)** | **<0.001** | **1.02 (1.01 - 1.04)** | **0.009** |
| Days within the same bay as the index case | | **0.93 (0.88 - 0.99)** | **0.021** | **0.96 (0.94 - 0.97)** | **<0.001** |
